# Genetically predicted circulating B vitamins in relation to digestive system cancers

**DOI:** 10.1038/s41416-021-01383-0

**Published:** 2021-04-09

**Authors:** Shuai Yuan, Paul Carter, Mathew Vithayathil, Siddhartha Kar, Amy M. Mason, Stephen Burgess, Susanna C. Larsson

**Affiliations:** 1 Unit of Cardiovascular and Nutritional Epidemiology, Institute of Environmental Medicine, Karolinska Institutet, Stockholm, Sweden; 2 Department of Medicine, University of Cambridge, Cambridge, UK; 3 MRC Cancer Unit, University of Cambridge, Cambridge, UK; 4 MRC Integrative Epidemiology Unit, Bristol Medical School, University of Bristol, Bristol, UK; 5 British Heart Foundation Cardiovascular Epidemiology Unit, Department of Public Health and Primary Care, University of Cambridge, Cambridge, UK; 6 National Institute for Health Research Cambridge Biomedical Research Centre, University of Cambridge and Cambridge University Hospitals, Cambridge, UK; 7 MRC Biostatistics Unit, University of Cambridge, Cambridge, UK; 8 Department of Public Health and Primary Care, University of Cambridge, Cambridge, UK; 9 Unit of Medical Epidemiology, Department of Surgical Sciences, Uppsala University, Uppsala, Sweden

**Keywords:** Predictive markers, Gastrointestinal cancer, Cancer genetics

## Abstract

**Background:**

Folate, vitamin B6 and vitamin B12 have been associated with digestive system cancers. We conducted a two-sample Mendelian randomisation study to assess the causality of these associations.

**Methods:**

Two, one and 14 independent single nucleotide polymorphisms associated with serum folate, vitamin B6 and vitamin B12 at the genome-wide significance threshold were selected as genetic instruments. Summary-level data for the associations of the vitamin-associated genetic variants with cancer were obtained from the UK Biobank study including 367,561 individuals and FinnGen consortium comprising up to 176,899 participants.

**Results:**

Genetically predicted folate and vitamin B6 concentrations were not associated with overall cancer, overall digestive system cancer or oesophageal, gastric, colorectal or pancreatic cancer. Genetically predicted vitamin B12 concentrations were positively associated with overall digestive system cancer (OR_SD_, 1.12; 95% CI 1.04, 1.21, *p* = 0.003) and colorectal cancer (OR_SD_ 1.16; 95% CI 1.06, 1.26, *p* = 0.001) in UK Biobank. Results for colorectal cancer were consistent in FinnGen and the combined OR_SD_ was 1.16 (95% CI 1.08, 1.25, *p* < 0.001). There was no association of genetically predicted vitamin B12 with any other site-specific digestive system cancers or overall cancer.

**Conclusions:**

These results provide evidence to suggest that elevated serum vitamin B12 concentrations are associated with colorectal cancer.

## Background

Folate, vitamin B6 and vitamin B12 have important roles in DNA methylation, synthesis and repair and have been proposed to modify the risk of cancer, in particular digestive system cancers. Notwithstanding, the association between these B vitamins and digestive system cancers is not fully understood. Findings on folate in relation to risk of colorectal cancer are conflicting with neutral,^1^ inverse^2^ and positive^3^ associations reported, whereas folate intake appears to lower the risk of upper gastrointestinal system cancers.^4,5^ Vitamin B6 and its principal active coenzyme form (pyridoxal 5′-phosphate) were identified to be associated with a reduced risk of all cancers and gastrointestinal cancers in a systematic review and meta-analysis involving 121 observational studies.^6^ Although randomised controlled trials (RCTs) have revealed no association between supplementation of B vitamins and overall cancer incidence, no trials to date have studied cancer as the primary endpoint or with sufficient incident cancers.^6^ Elevated plasma vitamin B12 concentrations have been associated with an increased cancer incidence within the first year of follow-up,^7,8^ but the long-term consequences of elevated vitamin B12 concentrations on the risk of overall digestive system cancer have been scarcely investigated and results are conflicting.^3,9–11^

Multivitamins and B vitamin supplements are commonly used in adults. Approximately 30–40% of U.S. adults take folate, vitamin B6 or vitamin B12 supplements, and the percentages are even higher among older Caucasian women.^12^ Meanwhile, cancer projects a heavy burden on human health worldwide.^13^ Thus, it is of great importance to understand the role of supplementation of B vitamins on cancer development.

Mendelian randomisation (MR) analysis can strengthen the causal inference in an exposure-outcome association by using genetic variants as instruments for an exposure.^14^ The MR design can minimise residual confounding and reverse causation biases.^14^ Here, we conducted an MR investigation to determine the associations of genetically predicted circulating concentrations of folate and of vitamins B6 and B12 concentrations with the risk of overall and digestive system cancers. Folate and vitamin B12 deficiency is associated with pernicious anaemia and mean corpuscular volume,^15^ which were used as positive control outcomes for the genetic instrument for folate and vitamin B12 concentrations (Supplementary method).

## Methods

### Genetic instrument selection

Three, one and 15 single nucleotide polymorphisms (SNPs) associated with serum folate, vitamin B6 and vitamin B12 at the genome-wide significance level (*p* < 5 × 10^−8^) were identified in meta-analyses of genome-wide association studies (GWASs) on folate (37,341 individuals of European ancestries),^16^ vitamin B6 (1864 individuals of European descent),^17^ and vitamin B12 (45,576 individuals of European ancestries).^16^ The average concentrations of B vitamins in these GWASs were ~20.0 nmol/L for folate, 9.4 ng/mL for vitamin B6 and 391.0 pmol/L for vitamin B12. The SNPs explained ~1.0%, 2.8% and 6.3% phenotypic variance for folate, vitamin B6 and vitamin B12, respectively. Linkage disequilibrium among SNPs for folate and vitamin B12 was estimated based on the 1000 Genomes European panel and SNPs with linkage disequilibrium (*r*^2^ > 0.01 or clumping distance>10 kb) were removed, leaving two and 14 SNPs as instrumental variables for serum folate and vitamin B12, respectively.

### Cancer data sources

We obtained summary-level data (beta coefficients and corresponding standard errors) for the associations of the B vitamin-associated SNPs with cancer from the UK Biobank study^18^ and the FinnGen consortium.^19^ In UK Biobank, there were 90,363 cases for overall cancer, 11,061 cases for digestive system cancer (including oesophageal, gastric, pancreatic, hepatocellular, biliary tract and colorectal cancers), 1228 cases for oesophageal cancer, 994 cases for gastric cancer, 6995 cases for colorectal cancer and 1747 cases for pancreatic cancer and up to 307,914 non-cancer cases. The FinnGen ascertained 29,617 cases for overall cancer, 4298 cases for digestive system cancer (similarly defined in UK Biobank), 193 cases for oesophageal cancer, 506 cases for gastric cancer, 2435 cases for colorectal cancer and 519 cases for pancreatic cancer and 147,282 non-cancer cases. Data sources descriptions, diagnostic information and used data are presented in Supplementary Methods and Supplementary Tables 1–3.

### Statistical analysis

The inverse-variance weighted method under a multiplicative random-effects model was employed as the primary statistical method.^20^ Estimates from UK Biobank and FinnGen were combined using fixed-effects meta-analysis. We performed several sensitivity analyses (Supplementary methods). The *I*^2^ statistic was calculated to assess the heterogeneity^21^ and the *p*-value for intercept in MR-Egger was used to assess the directional pleiotropy.^22^ Pleiotropic associations of the used SNPs with other traits were searched in PhenoScanner V2 (Supplementary Table 4).^23^ We scaled odds ratios (ORs) and corresponding 95% confidence intervals (CIs) to one standard deviation (SD) increase in serum folate, vitamin B6 and vitamin B12 concentrations. The Bonferroni method was used to correct for multiple testing, and associations with *p*-values < 0.008 (*p* = 0.05/6 outcomes) were regarded significant. All analyses were two-sided and performed using the mrrobust package^24^ in Stata/SE 15.0 and TwoSampleMR^25^ in R Software 3.6.0.

## Results

**Fig. 1:**
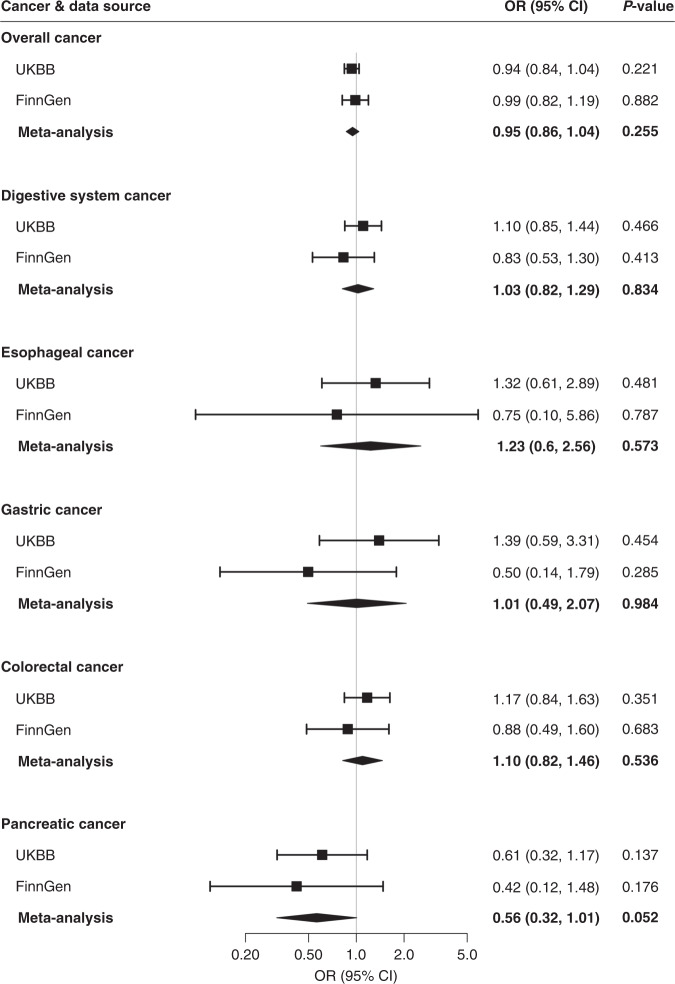
Associations of genetically predicted higher folate concentrations with cancer. CI indicates confidence interval, OR odds ratio, UKBB UK Biobank. Estimates were derived from the inverse-variance weighed method with random effects.

**Fig. 2:**
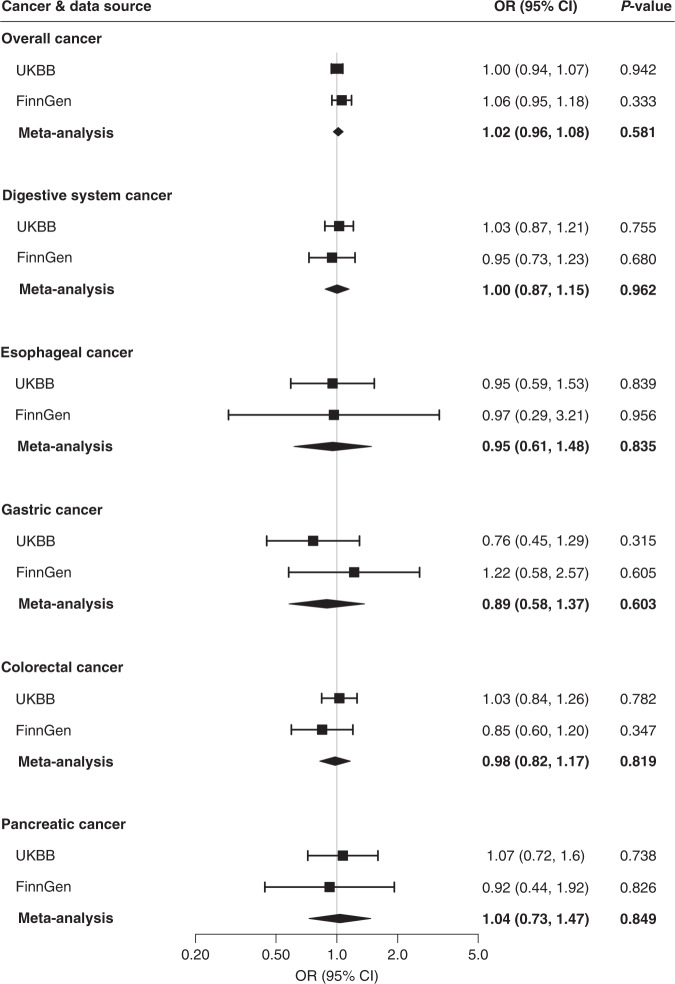
Associations of genetically predicted higher vitamin B6 concentrations with cancer. CI indicates confidence interval, OR odds ratio, UKBB UK Biobank. Estimates were derived from the inverse-variance weighed method with random effects.

**Fig. 3:**
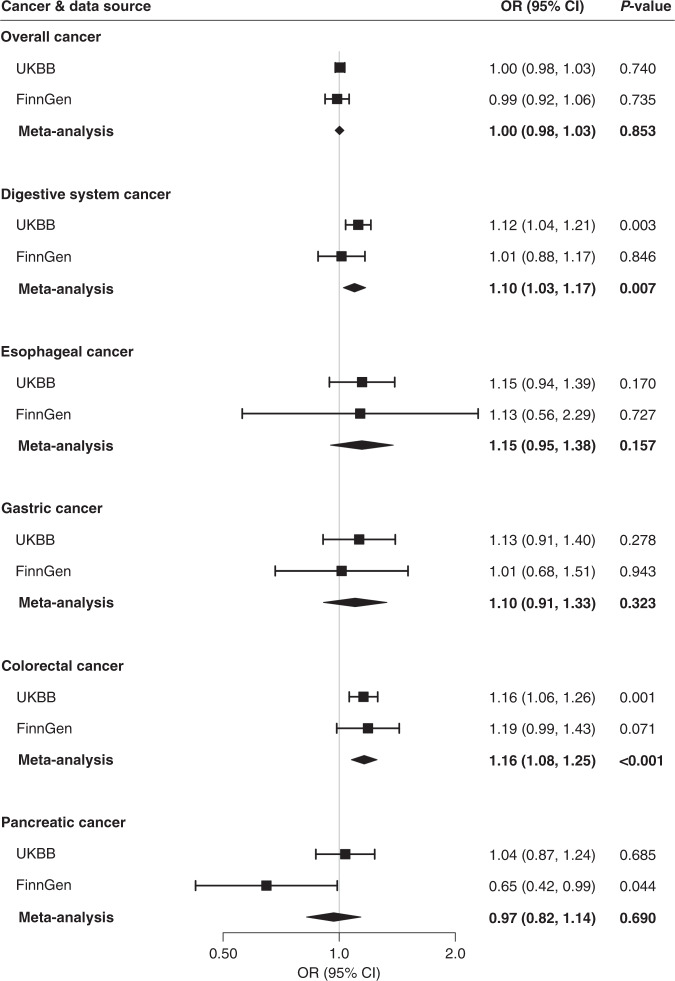
Associations of genetically predicted higher vitamin B12 concentrations with cancer. CI indicates confidence interval, OR odds ratio, UKBB UK Biobank. Estimates were derived from the inverse-variance weighed method with random effects.

Associations for positive controls are presented in Supplementary results and Supplementary Figs. 1, 2. Genetically predicted circulating concentrations of folate and vitamin B6 were not associated with risk of overall cancer, overall digestive system cancer or oesophageal, gastric, colorectal or pancreatic cancer in either UK Biobank or FinnGen (Figs. 1 and 2). Genetically predicted serum concentrations of vitamin B12 were associated with overall digestive system cancer risk in UK Biobank (OR_SD_ 1.12; 95% CI, 1.04, 1.21, *p* = 0.003) and in the meta-analysis (OR_SD_ 1.10; 95% CI, 1.03, 1.17, *p* = 0.007) after correction for multiple testing (Fig. 3). Genetically predicted high serum vitamin B12 was associated with an elevated risk of colorectal cancer in UK Biobank (OR_SD_ 1.16; 95% CI, 1.06, 1.26, *p* = 0.001) and both the direction and magnitude of the association remained in FinnGen (OR_SD_ 1.19; 95% CI, 0.99, 1.43, *p* = 0.071). The combined OR_SD_ of colorectal cancer was 1.16 (95% CI, 1.08, 1.25, *p* < 0.001) (Fig. 3). The associations of vitamin B12 with overall digestive system cancer and colorectal cancer were directionally consistent in sensitivity analyses (Table 1). We did not detect any SNPs driving the associations of vitamin B12 with overall digestive system cancer or colorectal cancer (Supplementary Fig. 3). Genetically predicted serum vitamin B12 was not associated with overall cancer, or oesophageal, gastric or pancreatic cancer risk (Fig. 3).

**Table 1:** Associations of genetically predicted higher vitamin B12 concentrations with cancer in sensitivity analyses.

Cancer	*I*^2^ (%)	*P* (*I*^2^)	Weighted median			MR-Egger			
			OR	95% CI	*p*	OR	95% CI	*p*	*p*_intercept_
Overall cancer (UKBB)	15	0.323	1.02	0.98–1.05	0.394	0.98	0.94–1.03	0.403	0.184
Overall cancer (FinnGen)	16	0.142	0.94	0.86–1.02	0.130	0.97	0.82–1.14	0.691	0.780
Digestive system cancer (UKBB)	17	0.207	1.06	0.97–1.16	0.192	1.00	0.90–1.11	0.955	0.007
Digestive system cancer (FinnGen)	9	0.630	1.00	0.83–1.21	0.987	1.04	0.77–1.41	0.805	0.859
Oesophageal (UKBB)	8	0.769	1.18	0.92–1.52	0.189	1.17	0.86–1.61	0.320	0.848
Oesophageal (FinnGen)	13	0.271	1.77	0.74–4.21	0.197	4.23	1.05–16.94	0.042	0.036
Gastric (UKBB)	10	0.672	1.03	0.78–1.36	0.846	0.89	0.63–1.27	0.526	0.098
Gastric (FinnGen)	11	0.458	0.91	0.52–1.58	0.727	0.86	0.36–2.10	0.748	0.691
Pancreatic (UKBB)	15	0.302	1.14	0.92–1.43	0.237	1.03	0.77–1.39	0.845	0.955
Pancreatic (FinnGen)	13	0.304	0.63	0.36–1.08	0.092	0.60	0.23–1.58	0.303	0.870
Colorectal (UKBB)	14	0.385	1.10	0.98–1.22	0.103	1.05	0.92–1.20	0.462	0.078
Colorectal (FinnGen)	3	0.984	1.22	0.96–1.54	0.100	1.20	0.80–1.80	0.381	0.961

*CI* confidence interval, *OR* odds ratio, *UKBB* UK Biobank.

The *I*^2^ (%) presents the heterogeneity among SNPs in each analysis and *the p*_intercept_ is the *p*-value for the intercept in MR-Egger.

## Discussion

The present MR study validated genetic instruments for folate and vitamin B12 using pernicious anaemia (for folate and vitamin B12) and mean corpuscular volume (for folate) as positive control outcomes and found that genetically predicted high concentrations of serum vitamin B12 were associated with an increased risk of overall digestive system cancer and colorectal cancer, but not overall cancer or oesophageal, gastric or pancreatic cancer. Genetically predicted serum folate and vitamin B6 concentrations were not significantly associated with overall cancer or digestive system cancers.

A pan-carcinogenic effect of high vitamin b12 was not found in our study, which is consistent with previous RCTs focusing on effects of administration of folic acid, vitamin B6 and vitamin B12 altogether.^26–28^ Nevertheless, a 21% higher risk of any cancer was previously observed with co-supplementation of folate and vitamin B12, when compared to placebo, after a median 39 months of treatment and an additional 38 months of post-trial observational follow-up in another RCT.^29^ Even though the influence of reverse causality is minimal in the RCT design, this positive finding might be related to an altered vitamin B12 metabolism caused by carcinogenesis prior to clinical cancer diagnosis.^7,8^

Data on vitamin B12 supplementation and gastrointestinal cancer are scarce and results on vitamin B12 in relation to colorectal cancer risk in observational studies are conflicting.^9,30^ One clinical trial reported an increased risk of colorectal cancer after combined folate and vitamin B12 supplementations,^3^ which may be considered support for our findings of positive associations of genetically predicted vitamin B12 with the digestive system and colorectal cancer. Furthermore, a previous MR study revealed a possible association between serum vitamin B12 and colorectal cancer albeit with concerns on potential pleiotropy.^31^ Our study confirmed this association in two independent populations (not included in previous MR study) and a series of statistical models minimising influence from pleiotropy. Given that colorectal cancer makes up a large proportion of digestive system cancer in UK Biobank it is possible that the observed association between serum vitamin B12 and digestive system cancer might be driven solely by its effect on colorectal cancer.

Rich sources of vitamin B12 include meat, which has previously been strongly associated with colorectal cancer risk and may therefore offer a mechanistic explanation for our findings.^32^ On a cellular level, vitamin B12 has a vital role in one-carbon metabolism that influences DNA synthesis, methylation as well as redox and reductive metabolism. Thus, vitamin B12 may influence pathways enhancing the proliferation of cancerous cells.^33^ It is relevant that the colorectal mucosa is the tissue with the highest turnover and is therefore particularly sensitive to nutrients such as B vitamins, which affect this process. A recent study demonstrated a higher serum B12 was associated with tumour-specific hypomethylation in CRC patients, suggestive of B12-driving epigenetic alterations leading to carcinogenesis.^34^ Also, of relevance, is that vitamin b12 can be produced by the gut microbiome and that competition of b12 may affect the gut microbiome, which offers a further putative mechanistic link with colorectal cancer.^35^ A possible carcinogenic effect of high serum vitamin B12 status has previously been observed in lung cancer^36^ and possibly epithelial ovarian cancer,^37^ but not in breast cancer.^38^ Furthermore, a recent MR study revealed a positive association between genetically predicted high serum vitamin B12 concentrations and risk of inflammatory bowel disease, especially Crohn’s disease.^39^ This may be of relevance as patients diagnosed with inflammatory bowel disease have an increased risk of developing colorectal cancer.^40^

Epidemiological data on folate (or folic acid) in relation to cancer are conflicting. Two meta-analyses based on RCTs found an increase in the frequency of overall cancer in the folic acid supplementation group compared to controls.^41,42^ A borderline increased overall cancer incidence was observed in a subsequent meta-analysis of RCTs including 50,000 individuals, although this finding was compatible with a chance finding as the risk did not increase with duration of treatment or a daily dose of folic acid.^43^ Another meta-analysis including 13 trials showed no association between folate intake and cancer risk,^1^ consistent with our finding. With regard to colorectal cancer specifically, an inverse association with total folate intake was observed in observational studies,^2^ although evidence from RCTs does not support an association between folate supplementation and colorectal cancer risk,^2^ which is in line with this MR study’s finding.

Dietary vitamin B6 intake showed inverse associations with any cancer, overall gastrointestinal cancer and oesophageal, gastric, colorectal^44^ and pancreatic cancers in meta-analyses of observational studies.^6^ However, several RCTs did not detect such associations.^6,26–28^ This discrepancy might be related to cancer-protective confounding effects from foods rich in vitamin B6, such as whole grains.^45^ Our study found null associations of vitamin B6 concentrations proxied by a single genetic variant with cancer risk and thus had offers limited support of any potential application of vitamin B6 in cancer prevention. We cannot rule out weak associations of vitamin B6 given small numbers of cases for some certain site-specific cancer and the use of only one variant, which explains a relatively low variance in vitamin b6 levels.

There are several strengths and limitations of the present study. The major strength is the MR design that reinforces the causal inference. In addition, we examined the associations in two independent populations and the consistency of results makes our findings robust. The combination of two independent data also increases our sample size, thereby enhancing the power to detect associations. However, the study may still overlook associations for certain site-specific cancers due to a small number of cases even after the combination of datasets, especially for folate and vitamin B6 with small variance explained by genetic instruments. Another strength of the present study is that most SNPs used as proxies for B vitamin concentrations are located in genes with known functions related to folate, vitamin B6 and vitamin B12 and their corresponding metabolic pathways.^46^ For folate and vitamin B12, we validated the genetic instruments using positive controls. The results of pleiotropic effect assessment and sensitivity analyses further confirm that our findings are less likely to be biased by pleiotropy. However, caution is required in interpreting the positive association between vitamin B12 and colorectal cancer in the absence of biological mechanistic evidence. Lastly, the population analyzed was confined to European individuals and thus population stratification bias was diminished.

This study also has important limitations. The population confinement to individuals of European descent limits the generalisability of our findings to other populations. Another limitation is that we cannot assess the gene-environment interaction using summary-level data. In this regard, some observational studies have found that the association of folate and vitamin B6 with cancer risk is modified by the intake of alcohol,^47–50^ which may interfere with the intestinal absorption and metabolism of these nutrients. An additional limitation is that our results for vitamin B6 were based on a single genetic variant identified in a GWAS of an Italian population and we were unable to validate that this variant is associated with vitamin B6 concentrations in the UK and Finnish populations. The analyses of folate and vitamin B6 were based on few SNPs, and therefore sensitivity analyses could not be performed. The associations of genetically predicted vitamin B12 with colorectal cancer and overall digestive system cancer in the sensitivity analyses were directionally consistent with the associations from the main analysis albeit nonsignificant, which might be caused by inadequate power.^51^ Further studies with larger power are needed to verify our findings.

In conclusion, the present study found that genetically predicted lifelong high serum vitamin B12 concentrations were associated with an increased risk of colorectal cancer. Further evidence is needed to assess the safety of wide use of vitamin B12 supplementation with respect to colorectal cancer development.

## Supplementary information

Supporting materials

## Data Availability

All data analyzed in this study are available in the supplemental material.
