# Supplementary material for: Genetically predicted circulating B vitamins in relation to digestive system cancers
Source: Br J Cancer. 2021 Apr 9;124(12):1997–2003. doi: 10.1038/s41416-021-01383-0 (PMC8184856; doi:10.1038/s41416-021-01383-0)

**Supporting materials**

**Genetically Predicted Circulating B Vitamins in Relation to Digestive System Cancers**

Shuai Yuan, Paul Carter, Mathew Vithayathil, Siddhartha Kar, Amy M. Mason, Stephen Burgess, Susanna C. Larsson

**Supplementary methods and results**

**Supplementary Table 1.** Diagnostic information of cancer in UK Biobank and FinnGen

**Supplementary Table 2.** Data used in the present study from UK Biobank

**Supplementary Table 3.** Data used in the present study from FinnGen

**Supplementary Table 4**. Diseases and traits associated with genetic variants identified for folate, vitamin B6, and vitamin B12 at the genome-wide significance level

**Supplementary Figure 1.** Associations of genetically predicted higher serum folate and vitamin B12 with pernicious anemia (positive control outcome)

**Supplementary Figure 2.** Associations of genetically predicted higher serum folate and vitamin B12 with mean corpuscular volume (positive control outcome)

**Supplementary Figure 3.** Associations of genetically predicted higher levels of vitamin B12 with any digestive system cancer and colorectal cancer in leave-one-out analysis based on UK Biobank

**Supplementary methods and results**

**UK Biobank**

The UK Biobank study recruited about 500 000 adults, aged 37 to 73 years, between 2006 and 2010.^1^ The present analysis included 367 561 individuals after exclusion of those with non-Caucasian ethnicity (to reduce population stratification bias), those related by third degree or higher, excess heterozygosity, and low genotype call rate. Cancer cases were defined based on data from national registries (International Classification of Diseases, 9th and 10th revision codes) and self-reported information verified by interview with a nurse and were obtained until June 30, 2020. Association tests were adjusted for age, sex and ten genetic principal components.

**FinnGen consortium**

The FinnGen consortium encompasses 176  899 Finnish individuals in the last publicly available (R4) data release.^2^ Individuals with ambiguous gender, high genotype missingness (>5%), excess heterozygosity (±4 standard deviation) and non-Finnish ancestry were excluded. Cancer cases were defined by International Classification of Diseases, 8th, 9th and 10th revision codes with information from nationwide registries. Genome-wide association tests adjusted for age, sex, the first ten genetic principal components, and genotyping batch.

**Ethical permit**

All included GWASs were approved by corresponding ethics committees and participants provided written informed consent. The present analyses were approved by the Swedish Ethical Review Authority.

**Positive control data sources**

Summary-level data for pernicious anemia were obtained from the Neale Lab’s results of genome-wide association analysis of UK Biobank data. In total, 1020 pernicious anemia cases and 336 139 non-cases were ascertained by self-reported information on diagnosis of pernicious anemia. Data were harmonized and extracted out via MR-Base platform.^3^ Summary-level data for mean corpuscular volume were available from a meta-analysis of GWASs including up to 563 946 individuals of European ancestry.^4^

**Sensitivity analyses**

The weighted median method provides consistent causal estimates assuming more than 50% of weight comes from valid instruments.^5^ The MR-Egger regression can detect and correct for directional pleiotropy but typically has low precision.^6^ We performed leave-one-out analysis for association with *p* value <0.05 in one outcome source to examine whether the association is driven by any particular single SNP. We also conducted sensitivity analyses excluding SNPs with pleiotropic effects that might bias the results.

**Associations with positive controls**

Genetically-predicted serum concentrations of folate (OR_SD_ 0.36; 95% CI, 0.15, 0.84, *p*=0.018) and vitamin B12 (OR_SD_ 0.47; 95% CI, 0.28, 0.81, *p*=0.006) showed inverse associations with pernicious anemia in the primary analysis and results were consistent in sensitivity analyses (**Supplementary Figure 1)**. Higher genetically-predicted folate (estimate_SD_ -0.12; 95% CI, -0.16, -0.09, *p*<0.001) but not vitamin B12 (estimate_SD_ -0.01; 95% CI, -0.03, 0.01, *p*=0.200) concentrations were associated with decreased levels of mean corpuscular volume (**Supplementary Figure 2)**.

**Pleiotropy assessment and correction**

We observed pleiotropic effects from blood pressure for rs1801133 (folate), liver enzymes for rs4654748 (vitamin B6), liver enzymes, blood cholesterol, urinary sodium, gallstone, Crohn’s disease and alcohol intake for rs34324219 (vitamin B12), Lewis system Lea antigen for rs602662 (vitamin B12), and cardiovascular disease for rs1131603 (vitamin B12). Results of pleiotropic effect assessment are presented in **Supplementary Table 4**. Pleiotropic effects of rs602662 and rs34324219 might bias the association between vitamin B12 and cancer^7,8^ and the removal of these SNPs generated only a limited change in results. For colorectal cancer, the combined OR_SD_s were 1.12 (95% CI, 1.03, 1.22, *p*=0.006) after exclusion of rs602662, 1.17 (95% CI, 1.08, 1.27, *p*<0.001) after exclusion of rs34324219, and 1.13 (95% CI, 1.03, 1.23, *p*=0.007) after exclusion of both.

**References**

1. Sudlow C, Gallacher J, Allen N, Beral V, Burton P, Danesh J *et al.* UK biobank: an open access resource for identifying the causes of a wide range of complex diseases of middle and old age. *PLoS Med* 2015; **12**(3): e1001779; e-pub ahead of print 2015/04/01; doi 10.1371/journal.pmed.1001779.

2. consortium F. FinnGen documentation of R3 release, 2020. https://finngen.gitbook.io/documentation/. Accessed 28 July, 2020 (2020).

3. Hemani G, Zheng J, Elsworth B, Wade KH, Haberland V, Baird D *et al.* The MR-Base platform supports systematic causal inference across the human phenome. *Elife* 2018; **7**; e-pub ahead of print 2018/05/31; doi 10.7554/eLife.34408.

4. Chen MH, Raffield LM, Mousas A, Sakaue S, Huffman JE, Moscati A *et al.* Trans-ethnic and Ancestry-Specific Blood-Cell Genetics in 746,667 Individuals from 5 Global Populations. *Cell* 2020; **182**(5): 1198-1213.e1114; e-pub ahead of print 2020/09/06; doi 10.1016/j.cell.2020.06.045.

5. Bowden J, Davey Smith G, Haycock PC, Burgess S. Consistent Estimation in Mendelian Randomization with Some Invalid Instruments Using a Weighted Median Estimator. *Genet Epidemiol* 2016; **40**(4): 304-314; e-pub ahead of print 2016/04/12; doi 10.1002/gepi.21965.

6. Bowden J, Davey Smith G, Burgess S. Mendelian randomization with invalid instruments: effect estimation and bias detection through Egger regression. *Int J Epidemiol* 2015; **44**(2): 512-525; e-pub ahead of print 2015/06/08; doi 10.1093/ije/dyv080.

7. Gillen CD, Walmsley RS, Prior P, Andrews HA, Allan RN. Ulcerative colitis and Crohn's disease: a comparison of the colorectal cancer risk in extensive colitis. *Gut* 1994; **35**(11): 1590-1592; e-pub ahead of print 1994/11/01; doi 10.1136/gut.35.11.1590.

8. Torrado J, Blasco E, Gutierrez-Hoyos A, Cosme A, Lojendio M, Arenas JI. Lewis system alterations in gastric carcinogenesis. *Cancer* 1990; **66**(8): 1769-1774; e-pub ahead of print 1990/10/15; doi 10.1002/1097-0142(19901015)66:8<1769::aid-cncr2820660822>3.0.co;2-q.

**Supplementary Table 1.** Diagnostic information of cancer in UK Biobank and FinnGen

| **Cancer site/cancer** | **Diagnostic information** | | |
| --- | --- | --- | --- |
| **UK Biobank** | **ICD-9 codes** | **ICD-10 codes** | **Self-reported cancer** |
| Digestive system cancer | 150, V10.03, 151, V10.04, 157, 155, 156, 153, 154.0, 154.1, V10.05, V10.06 | C15, Z85.01, C16, Z85.028, C25, Z85.07, C22.0, C22.1, C23.X, C24.X, C18, C19, C20, Z85.038, Z85.048 | 1017, 1018, 1034, 1024, 1025, 1020, 1022, 1023 |
| Esophagus | 150, V10.03 | C15, Z85.01 | 1017 |
| Stomach | 151, V10.04 | C16, Z85.028 | 1018 |
| Colorectum | 153, 154.0, 154.1, V10.05, V10.06 | C18, C19, C20, Z85.038, Z85.048 | 1020, 1022, 1023 |
| Pancreas | 157 | C25, Z85.07 | 1034 |
| **FinnGen** | **ICD-8 codes** | **ICD-9 codes** | **ICD-10 codes** |
| Digestive system cancer | 150, 151, 152, 153, 1540, 1541, 1542, 155, 1560, 156, 157, 158, 159 | 150, 151, 152, 153, 154, 155, 156, 157, 158, 159 | C15, C16, C17, C18, C19, C20, C21, C22, C23, C24, C25, C26 |
| Esophagus | 150 | 150 | C15 |
| Stomach | 151 | 151 | C16 |
| Colorectum | 153, 1540, 1541 | 153, 154 | C18, C19, C20 |
| Pancreas | 157 | 157 | C25 |

**Supplementary Table 2.** Data used in the present study from UK Biobank

|  |  |  |  |  |  | **B vitamin** | | | **Outcome** | | |
| --- | --- | --- | --- | --- | --- | --- | --- | --- | --- | --- | --- |
| **Outcome** | **Vitamin** | **SNP** | **Gene** | **EA** | **NEA** | **Beta** | **SE** | **p** | **Beta** | **SE** | **p** |
| All_cancer | B12 | rs12272669 | MMACHC | A | G | 0.510 | 0.007 | 3.00E-09 | 0.008 | 0.011 | 0.431 |
| All_cancer | B12 | rs2270655 | MMAA | G | C | 0.066 | 0.018 | 2.20E-13 | 0.001 | 0.013 | 0.936 |
| All_cancer | B12 | rs1141321 | MUT | C | T | 0.061 | 0.007 | 3.60E-26 | 0.006 | 0.006 | 0.334 |
| All_cancer | B12 | rs1801222 | CUBN | G | A | 0.110 | 0.007 | 3.30E-75 | -0.001 | 0.006 | 0.812 |
| All_cancer | B12 | rs117456053 | TCN1 | G | A | 0.160 | 0.026 | 1.90E-09 | -0.022 | 0.026 | 0.401 |
| All_cancer | B12 | rs34324219 | TCN1 | C | A | 0.210 | 0.007 | 1.10E-111 | -0.012 | 0.009 | 0.175 |
| All_cancer | B12 | rs34528912 | TCN1 | T | C | 0.170 | 0.021 | 2.10E-15 | 0.007 | 0.014 | 0.634 |
| All_cancer | B12 | rs56077122 | CUBN/TRDMT1 | A | C | 0.087 | 0.009 | 4.80E-21 | 0.007 | 0.006 | 0.226 |
| All_cancer | B12 | rs41281112 | CLYBL | C | T | 0.170 | 0.020 | 8.90E-35 | -0.014 | 0.017 | 0.402 |
| All_cancer | B12 | rs3742801 | ABCD4 | T | C | 0.045 | 0.009 | 1.70E-13 | 0.014 | 0.006 | 0.019 |
| All_cancer | B12 | rs2336573 | CD320 | T | C | 0.320 | 0.007 | 8.40E-59 | 0.002 | 0.015 | 0.886 |
| All_cancer | B12 | rs602662 | FUT2 | A | G | 0.160 | 0.007 | 2.40E-139 | -0.007 | 0.006 | 0.238 |
| All_cancer | B12 | rs7788053 | FUT6 | A | G | 0.046 | 0.007 | 1.70E-10 | 0.257 | 0.792 | 0.746 |
| All_cancer | B12 | rs1131603 | TCN2 | C | T | 0.190 | 0.017 | 4.90E-49 | 0.015 | 0.013 | 0.253 |
| All_cancer | B6 | rs4654748 | ALPL | T | C | 1.450 | 0.280 | 8.30E-18 | 0.000 | 0.006 | 0.942 |
| All_cancer | Folate | rs1801133 | MTHFR | G | A | 0.096 | 0.008 | 9.50E-53 | -0.009 | 0.006 | 0.127 |
| All_cancer | Folate | rs652197 | FOLR3 | C | T | 0.069 | 0.011 | 1.40E-12 | 0.002 | 0.008 | 0.764 |
| Colorectum | B12 | rs12272669 | MMACHC | A | G | 0.510 | 0.007 | 3.00E-09 | 0.044 | 0.033 | 0.179 |
| Colorectum | B12 | rs2270655 | MMAA | G | C | 0.066 | 0.018 | 2.20E-13 | 0.029 | 0.039 | 0.451 |
| Colorectum | B12 | rs1141321 | MUT | C | T | 0.061 | 0.007 | 3.60E-26 | 0.041 | 0.018 | 0.022 |
| Colorectum | B12 | rs1801222 | CUBN | G | A | 0.110 | 0.007 | 3.30E-75 | 0.014 | 0.018 | 0.445 |
| Colorectum | B12 | rs117456053 | TCN1 | G | A | 0.160 | 0.026 | 1.90E-09 | -0.061 | 0.082 | 0.460 |
| Colorectum | B12 | rs34324219 | TCN1 | C | A | 0.210 | 0.007 | 1.10E-111 | 0.005 | 0.028 | 0.867 |
| Colorectum | B12 | rs34528912 | TCN1 | T | C | 0.170 | 0.021 | 2.10E-15 | 0.051 | 0.044 | 0.250 |
| Colorectum | B12 | rs56077122 | CUBN/TRDMT1 | A | C | 0.087 | 0.009 | 4.80E-21 | -0.004 | 0.019 | 0.810 |
| Colorectum | B12 | rs41281112 | CLYBL | C | T | 0.170 | 0.020 | 8.90E-35 | -0.001 | 0.053 | 0.982 |
| Colorectum | B12 | rs3742801 | ABCD4 | T | C | 0.045 | 0.009 | 1.70E-13 | 0.020 | 0.018 | 0.259 |
| Colorectum | B12 | rs2336573 | CD320 | T | C | 0.320 | 0.007 | 8.40E-59 | 0.025 | 0.046 | 0.593 |
| Colorectum | B12 | rs602662 | FUT2 | A | G | 0.160 | 0.007 | 2.40E-139 | 0.061 | 0.017 | 0.000 |
| Colorectum | B12 | rs7788053 | FUT6 | A | G | 0.046 | 0.007 | 1.70E-10 | -1.033 | 1.557 | 0.507 |
| Colorectum | B12 | rs1131603 | TCN2 | C | T | 0.190 | 0.017 | 4.90E-49 | 0.045 | 0.041 | 0.274 |
| Colorectum | B6 | rs4654748 | ALPL | T | C | 1.450 | 0.280 | 8.30E-18 | 0.005 | 0.017 | 0.782 |
| Colorectum | Folate | rs1801133 | MTHFR | G | A | 0.096 | 0.008 | 9.50E-53 | 0.020 | 0.018 | 0.275 |
| Colorectum | Folate | rs652197 | FOLR3 | C | T | 0.069 | 0.011 | 1.40E-12 | -0.002 | 0.026 | 0.927 |
| Esophagus | B12 | rs12272669 | MMACHC | A | G | 0.510 | 0.007 | 3.00E-09 | 0.077 | 0.078 | 0.322 |
| Esophagus | B12 | rs2270655 | MMAA | G | C | 0.066 | 0.018 | 2.20E-13 | 0.014 | 0.091 | 0.875 |
| Esophagus | B12 | rs1141321 | MUT | C | T | 0.061 | 0.007 | 3.60E-26 | 0.020 | 0.042 | 0.631 |
| Esophagus | B12 | rs1801222 | CUBN | G | A | 0.110 | 0.007 | 3.30E-75 | -0.023 | 0.042 | 0.584 |
| Esophagus | B12 | rs117456053 | TCN1 | G | A | 0.160 | 0.026 | 1.90E-09 | -0.106 | 0.193 | 0.585 |
| Esophagus | B12 | rs34324219 | TCN1 | C | A | 0.210 | 0.007 | 1.10E-111 | 0.007 | 0.065 | 0.919 |
| Esophagus | B12 | rs34528912 | TCN1 | T | C | 0.170 | 0.021 | 2.10E-15 | -0.177 | 0.105 | 0.091 |
| Esophagus | B12 | rs56077122 | CUBN/TRDMT1 | A | C | 0.087 | 0.009 | 4.80E-21 | 0.067 | 0.044 | 0.131 |
| Esophagus | B12 | rs41281112 | CLYBL | C | T | 0.170 | 0.020 | 8.90E-35 | -0.018 | 0.126 | 0.885 |
| Esophagus | B12 | rs3742801 | ABCD4 | T | C | 0.045 | 0.009 | 1.70E-13 | -0.022 | 0.042 | 0.607 |
| Esophagus | B12 | rs2336573 | CD320 | T | C | 0.320 | 0.007 | 8.40E-59 | 0.111 | 0.110 | 0.310 |
| Esophagus | B12 | rs602662 | FUT2 | A | G | 0.160 | 0.007 | 2.40E-139 | 0.039 | 0.041 | 0.333 |
| Esophagus | B12 | rs1131603 | TCN2 | C | T | 0.190 | 0.017 | 4.90E-49 | 0.090 | 0.098 | 0.359 |
| Esophagus | B6 | rs4654748 | ALPL | T | C | 1.450 | 0.280 | 8.30E-18 | -0.008 | 0.040 | 0.839 |
| Esophagus | Folate | rs1801133 | MTHFR | G | A | 0.096 | 0.008 | 9.50E-53 | 0.064 | 0.043 | 0.134 |
| Esophagus | Folate | rs652197 | FOLR3 | C | T | 0.069 | 0.011 | 1.40E-12 | -0.085 | 0.061 | 0.163 |
| GI_cancer | B12 | rs12272669 | MMACHC | A | G | 0.510 | 0.007 | 3.00E-09 | 0.028 | 0.026 | 0.281 |
| GI_cancer | B12 | rs2270655 | MMAA | G | C | 0.066 | 0.018 | 2.20E-13 | 0.036 | 0.031 | 0.250 |
| GI_cancer | B12 | rs1141321 | MUT | C | T | 0.061 | 0.007 | 3.60E-26 | 0.048 | 0.014 | 0.001 |
| GI_cancer | B12 | rs1801222 | CUBN | G | A | 0.110 | 0.007 | 3.30E-75 | 0.005 | 0.014 | 0.723 |
| GI_cancer | B12 | rs117456053 | TCN1 | G | A | 0.160 | 0.026 | 1.90E-09 | 0.036 | 0.066 | 0.586 |
| GI_cancer | B12 | rs34324219 | TCN1 | C | A | 0.210 | 0.007 | 1.10E-111 | -0.003 | 0.022 | 0.898 |
| GI_cancer | B12 | rs34528912 | TCN1 | T | C | 0.170 | 0.021 | 2.10E-15 | 0.024 | 0.036 | 0.503 |
| GI_cancer | B12 | rs56077122 | CUBN/TRDMT1 | A | C | 0.087 | 0.009 | 4.80E-21 | 0.010 | 0.015 | 0.510 |
| GI_cancer | B12 | rs41281112 | CLYBL | C | T | 0.170 | 0.020 | 8.90E-35 | 0.028 | 0.043 | 0.519 |
| GI_cancer | B12 | rs3742801 | ABCD4 | T | C | 0.045 | 0.009 | 1.70E-13 | 0.019 | 0.014 | 0.177 |
| GI_cancer | B12 | rs2336573 | CD320 | T | C | 0.320 | 0.007 | 8.40E-59 | 0.020 | 0.037 | 0.596 |
| GI_cancer | B12 | rs602662 | FUT2 | A | G | 0.160 | 0.007 | 2.40E-139 | 0.039 | 0.014 | 0.005 |
| GI_cancer | B12 | rs7788053 | FUT6 | A | G | 0.046 | 0.007 | 1.70E-10 | -1.063 | 1.374 | 0.439 |
| GI_cancer | B12 | rs1131603 | TCN2 | C | T | 0.190 | 0.017 | 4.90E-49 | 0.050 | 0.033 | 0.134 |
| GI_cancer | B6 | rs4654748 | ALPL | T | C | 1.450 | 0.280 | 8.30E-18 | 0.004 | 0.014 | 0.755 |
| GI_cancer | Folate | rs1801133 | MTHFR | G | A | 0.096 | 0.008 | 9.50E-53 | 0.018 | 0.015 | 0.214 |
| GI_cancer | Folate | rs652197 | FOLR3 | C | T | 0.069 | 0.011 | 1.40E-12 | -0.017 | 0.021 | 0.406 |
| Pancreas | B12 | rs12272669 | MMACHC | A | G | 0.510 | 0.007 | 3.00E-09 | 0.075 | 0.065 | 0.244 |
| Pancreas | B12 | rs2270655 | MMAA | G | C | 0.066 | 0.018 | 2.20E-13 | 0.082 | 0.077 | 0.285 |
| Pancreas | B12 | rs1141321 | MUT | C | T | 0.061 | 0.007 | 3.60E-26 | 0.030 | 0.035 | 0.400 |
| Pancreas | B12 | rs1801222 | CUBN | G | A | 0.110 | 0.007 | 3.30E-75 | -0.016 | 0.035 | 0.639 |
| Pancreas | B12 | rs117456053 | TCN1 | G | A | 0.160 | 0.026 | 1.90E-09 | 0.278 | 0.162 | 0.086 |
| Pancreas | B12 | rs34324219 | TCN1 | C | A | 0.210 | 0.007 | 1.10E-111 | -0.028 | 0.055 | 0.614 |
| Pancreas | B12 | rs34528912 | TCN1 | T | C | 0.170 | 0.021 | 2.10E-15 | -0.018 | 0.088 | 0.841 |
| Pancreas | B12 | rs56077122 | CUBN/TRDMT1 | A | C | 0.087 | 0.009 | 4.80E-21 | 0.008 | 0.037 | 0.823 |
| Pancreas | B12 | rs41281112 | CLYBL | C | T | 0.170 | 0.020 | 8.90E-35 | 0.246 | 0.106 | 0.020 |
| Pancreas | B12 | rs3742801 | ABCD4 | T | C | 0.045 | 0.009 | 1.70E-13 | 0.010 | 0.035 | 0.780 |
| Pancreas | B12 | rs2336573 | CD320 | T | C | 0.320 | 0.007 | 8.40E-59 | -0.054 | 0.092 | 0.559 |
| Pancreas | B12 | rs602662 | FUT2 | A | G | 0.160 | 0.007 | 2.40E-139 | -0.055 | 0.034 | 0.107 |
| Pancreas | B12 | rs7788053 | FUT6 | A | G | 0.046 | 0.007 | 1.70E-10 | -0.980 | 3.966 | 0.805 |
| Pancreas | B12 | rs1131603 | TCN2 | C | T | 0.190 | 0.017 | 4.90E-49 | 0.025 | 0.082 | 0.758 |
| Pancreas | B6 | rs4654748 | ALPL | T | C | 1.450 | 0.280 | 8.30E-18 | 0.011 | 0.034 | 0.738 |
| Pancreas | Folate | rs1801133 | MTHFR | G | A | 0.096 | 0.008 | 9.50E-53 | -0.056 | 0.036 | 0.120 |
| Pancreas | Folate | rs652197 | FOLR3 | C | T | 0.069 | 0.011 | 1.40E-12 | -0.011 | 0.051 | 0.826 |
| Stomach | B12 | rs12272669 | MMACHC | A | G | 0.510 | 0.007 | 3.00E-09 | -0.025 | 0.085 | 0.767 |
| Stomach | B12 | rs2270655 | MMAA | G | C | 0.066 | 0.018 | 2.20E-13 | 0.180 | 0.102 | 0.076 |
| Stomach | B12 | rs1141321 | MUT | C | T | 0.061 | 0.007 | 3.60E-26 | 0.068 | 0.047 | 0.147 |
| Stomach | B12 | rs1801222 | CUBN | G | A | 0.110 | 0.007 | 3.30E-75 | 0.007 | 0.046 | 0.880 |
| Stomach | B12 | rs117456053 | TCN1 | G | A | 0.160 | 0.026 | 1.90E-09 | -0.190 | 0.215 | 0.377 |
| Stomach | B12 | rs34324219 | TCN1 | C | A | 0.210 | 0.007 | 1.10E-111 | -0.051 | 0.073 | 0.482 |
| Stomach | B12 | rs34528912 | TCN1 | T | C | 0.170 | 0.021 | 2.10E-15 | 0.012 | 0.116 | 0.921 |
| Stomach | B12 | rs56077122 | CUBN/TRDMT1 | A | C | 0.087 | 0.009 | 4.80E-21 | 0.021 | 0.049 | 0.669 |
| Stomach | B12 | rs41281112 | CLYBL | C | T | 0.170 | 0.020 | 8.90E-35 | 0.009 | 0.140 | 0.951 |
| Stomach | B12 | rs3742801 | ABCD4 | T | C | 0.045 | 0.009 | 1.70E-13 | 0.026 | 0.047 | 0.573 |
| Stomach | B12 | rs2336573 | CD320 | T | C | 0.320 | 0.007 | 8.40E-59 | 0.159 | 0.122 | 0.191 |
| Stomach | B12 | rs602662 | FUT2 | A | G | 0.160 | 0.007 | 2.40E-139 | 0.051 | 0.045 | 0.258 |
| Stomach | B12 | rs7788053 | FUT6 | A | G | 0.046 | 0.007 | 1.70E-10 | -0.995 | 5.438 | 0.855 |
| Stomach | B12 | rs1131603 | TCN2 | C | T | 0.190 | 0.017 | 4.90E-49 | 0.122 | 0.108 | 0.261 |
| Stomach | B6 | rs4654748 | ALPL | T | C | 1.450 | 0.280 | 8.30E-18 | -0.045 | 0.045 | 0.315 |
| Stomach | Folate | rs1801133 | MTHFR | G | A | 0.096 | 0.008 | 9.50E-53 | 0.042 | 0.048 | 0.377 |
| Stomach | Folate | rs652197 | FOLR3 | C | T | 0.069 | 0.011 | 1.40E-12 | -0.006 | 0.067 | 0.932 |
| Anemia | B12 | rs2270655 | MMAA | G | C | 0.066 | 0.018 | 1.23E-04 | -0.252 | 0.099 | 0.011 |
| Anemia | B12 | rs1141321 | MUT | C | T | 0.061 | 0.007 | 1.46E-18 | -0.098 | 0.045 | 0.031 |
| Anemia | B12 | rs1801222 | CUBN | G | A | 0.110 | 0.007 | 0.00E+00 | -0.175 | 0.045 | 0.000 |
| Anemia | B12 | rs56077122 | CUBN/TRDMT1 | A | C | 0.087 | 0.009 | 2.09E-22 | -0.070 | 0.048 | 0.141 |
| Anemia | B12 | rs117456053 | TCN1 | G | A | 0.160 | 0.026 | 3.78E-10 | -0.808 | 0.204 | 0.000 |
| Anemia | B12 | rs12272669 | MMACHC | A | G | 0.510 | 0.007 | 0.00E+00 | -0.026 | 0.080 | 0.741 |
| Anemia | B12 | rs34324219 | TCN1 | C | A | 0.210 | 0.007 | 0.00E+00 | -0.392 | 0.071 | 0.000 |
| Anemia | B12 | rs34528912 | TCN1 | T | C | 0.170 | 0.021 | 2.86E-16 | 0.288 | 0.113 | 0.011 |
| Anemia | B12 | rs41281112 | CLYBL | C | T | 0.170 | 0.020 | 9.48E-18 | -0.451 | 0.136 | 0.001 |
| Anemia | B12 | rs3742801 | ABCD4 | T | C | 0.045 | 0.009 | 2.87E-07 | -0.136 | 0.046 | 0.003 |
| Anemia | B12 | rs2336573 | CD320 | T | C | 0.320 | 0.007 | 0.00E+00 | -0.327 | 0.120 | 0.006 |
| Anemia | B12 | rs602662 | FUT2 | A | G | 0.160 | 0.007 | 0.00E+00 | -0.153 | 0.044 | 0.000 |
| Anemia | B12 | rs1131603 | TCN2 | C | T | 0.190 | 0.017 | 2.66E-29 | -0.360 | 0.105 | 0.001 |
| Anemia | Folate | rs1801133 | MTHFR | G | A | 0.096 | 0.008 | 9.50E-53 | -0.114 | 0.047 | 0.014 |
| Anemia | Folate | rs652197 | FOLR3 | C | T | 0.069 | 0.011 | 1.40E-12 | -0.027 | 0.066 | 0.677 |
| MCV | B12 | rs2270655 | MMAA | G | C | 0.066 | 0.018 | 2.20E-13 | 0.000 | 0.004 | 0.965 |
| MCV | B12 | rs1141321 | MUT | C | T | 0.061 | 0.007 | 3.60E-26 | 0.004 | 0.002 | 0.028 |
| MCV | B12 | rs7788053 | FUT6 | A | G | 0.046 | 0.007 | 1.70E-10 | 0.235 | 0.219 | 0.282 |
| MCV | B12 | rs1801222 | CUBN | G | A | 0.110 | 0.007 | 3.30E-75 | -0.001 | 0.002 | 0.531 |
| MCV | B12 | rs56077122 | CUBN/TRDMT1 | A | C | 0.087 | 0.009 | 4.80E-21 | -0.009 | 0.002 | 0.000 |
| MCV | B12 | rs117456053 | TCN1 | G | A | 0.160 | 0.026 | 1.90E-09 | -0.004 | 0.009 | 0.645 |
| MCV | B12 | rs12272669 | MMACHC | A | G | 0.510 | 0.007 | 3.00E-09 | 0.000 | 0.004 | 0.904 |
| MCV | B12 | rs34324219 | TCN1 | C | A | 0.210 | 0.007 | 1.10E-111 | -0.003 | 0.003 | 0.239 |
| MCV | B12 | rs34528912 | TCN1 | T | C | 0.170 | 0.021 | 2.10E-15 | -0.003 | 0.005 | 0.462 |
| MCV | B12 | rs41281112 | CLYBL | C | T | 0.170 | 0.020 | 8.90E-35 | -0.007 | 0.006 | 0.201 |
| MCV | B12 | rs3742801 | ABCD4 | T | C | 0.045 | 0.009 | 1.70E-13 | 0.007 | 0.002 | 0.000 |
| MCV | B12 | rs2336573 | CD320 | T | C | 0.320 | 0.007 | 8.40E-59 | 0.001 | 0.005 | 0.885 |
| MCV | B12 | rs602662 | FUT2 | A | G | 0.160 | 0.007 | 2.40E-139 | -0.009 | 0.002 | 0.000 |
| MCV | B12 | rs1131603 | TCN2 | C | T | 0.190 | 0.017 | 4.90E-49 | 0.001 | 0.004 | 0.813 |
| MCV | Folate | rs1801133 | MTHFR | G | A | 0.096 | 0.008 | 9.50E-53 | -0.015 | 0.002 | 0.000 |
| MCV | Folate | rs652197 | FOLR3 | C | T | 0.069 | 0.011 | 1.40E-12 | 0.002 | 0.003 | 0.555 |

EA indicates effect allele; NEA, non-effect allele; SE, standard error; SNP, single nucleotide polymorphism; VB, vitamin B.

**Supplementary Table 3.** Data used in the present study from FinnGen

|  |  |  |  |  |  | **B vitamin** | | | **Cancer** | | |
| --- | --- | --- | --- | --- | --- | --- | --- | --- | --- | --- | --- |
| **Cancer type** | **Vitamin** | **SNP** | **Gene** | **EA** | **NEA** | **Beta** | **SE** | ***p*** | **Beta** | **SE** | ***p*** |
| All_Cancer | B12 | rs2270655 | MMAA | G | C | 0.066 | 0.018 | 2.20E-13 | 0.002 | 0.020 | 0.930 |
| All_Cancer | B12 | rs1141321 | MUT | C | T | 0.061 | 0.007 | 3.60E-26 | 0.001 | 0.010 | 0.887 |
| All_Cancer | B12 | rs1801222 | CUBN | G | A | 0.110 | 0.007 | 0.00E+00 | -0.027 | 0.010 | 0.009 |
| All_Cancer | B12 | rs117456053 | TCN1 | G | A | 0.160 | 0.026 | 1.90E-09 | 0.022 | 0.030 | 0.458 |
| All_Cancer | B12 | rs34324219 | TCN1 | C | A | 0.210 | 0.007 | 0.00E+00 | 0.017 | 0.016 | 0.271 |
| All_Cancer | B12 | rs34528912 | TCN1 | T | C | 0.170 | 0.021 | 2.10E-15 | 0.004 | 0.021 | 0.853 |
| All_Cancer | B12 | rs56077122 | CUBN/TRDMT1 | A | C | 0.087 | 0.009 | 4.80E-21 | 0.023 | 0.011 | 0.041 |
| All_Cancer | B12 | rs41281112 | CLYBL | C | T | 0.170 | 0.020 | 8.90E-35 | -0.012 | 0.027 | 0.666 |
| All_Cancer | B12 | rs3742801 | ABCD4 | T | C | 0.045 | 0.009 | 1.70E-13 | 0.007 | 0.010 | 0.487 |
| All_Cancer | B12 | rs2336573 | CD320 | T | C | 0.320 | 0.007 | 0.00E+00 | 0.018 | 0.030 | 0.541 |
| All_Cancer | B12 | rs602662 | FUT2 | A | G | 0.160 | 0.007 | 0.00E+00 | -0.011 | 0.010 | 0.244 |
| All_Cancer | B12 | rs1131603 | TCN2 | C | T | 0.190 | 0.017 | 0.00E+00 | -0.015 | 0.017 | 0.361 |
| All_Cancer | B6 | rs4654748 | ALPL | T | C | 1.450 | 0.280 | 8.30E-18 | 0.009 | 0.010 | 0.332 |
| All_Cancer | Folate | rs1801133 | MTHFR | G | A | 0.096 | 0.008 | 0.00E+00 | -0.018 | 0.011 | 0.107 |
| All_Cancer | Folate | rs652197 | FOLR3 | C | T | 0.069 | 0.011 | 1.40E-12 | 0.026 | 0.012 | 0.032 |
| GI_Cancer | B12 | rs2270655 | MMAA | G | C | 0.066 | 0.018 | 2.20E-13 | 0.018 | 0.048 | 0.701 |
| GI_Cancer | B12 | rs1141321 | MUT | C | T | 0.061 | 0.007 | 3.60E-26 | -0.018 | 0.023 | 0.442 |
| GI_Cancer | B12 | rs1801222 | CUBN | G | A | 0.110 | 0.007 | 0.00E+00 | -0.029 | 0.024 | 0.222 |
| GI_Cancer | B12 | rs117456053 | TCN1 | G | A | 0.160 | 0.026 | 1.90E-09 | 0.005 | 0.070 | 0.944 |
| GI_Cancer | B12 | rs34324219 | TCN1 | C | A | 0.210 | 0.007 | 0.00E+00 | 0.000 | 0.036 | 0.999 |
| GI_Cancer | B12 | rs34528912 | TCN1 | T | C | 0.170 | 0.021 | 2.10E-15 | -0.063 | 0.050 | 0.210 |
| GI_Cancer | B12 | rs56077122 | CUBN/TRDMT1 | A | C | 0.087 | 0.009 | 4.80E-21 | 0.026 | 0.026 | 0.309 |
| GI_Cancer | B12 | rs41281112 | CLYBL | C | T | 0.170 | 0.020 | 8.90E-35 | -0.029 | 0.064 | 0.644 |
| GI_Cancer | B12 | rs3742801 | ABCD4 | T | C | 0.045 | 0.009 | 1.70E-13 | 0.016 | 0.024 | 0.517 |
| GI_Cancer | B12 | rs2336573 | CD320 | T | C | 0.320 | 0.007 | 0.00E+00 | 0.032 | 0.070 | 0.653 |
| GI_Cancer | B12 | rs602662 | FUT2 | A | G | 0.160 | 0.007 | 0.00E+00 | -0.001 | 0.023 | 0.975 |
| GI_Cancer | B12 | rs1131603 | TCN2 | C | T | 0.190 | 0.017 | 0.00E+00 | 0.072 | 0.040 | 0.071 |
| GI_Cancer | B6 | rs4654748 | ALPL | T | C | 1.450 | 0.280 | 8.30E-18 | -0.009 | 0.022 | 0.680 |
| GI_Cancer | Folate | rs1801133 | MTHFR | G | A | 0.096 | 0.008 | 0.00E+00 | -0.015 | 0.027 | 0.582 |
| GI_Cancer | Folate | rs652197 | FOLR3 | C | T | 0.069 | 0.011 | 1.40E-12 | -0.018 | 0.028 | 0.519 |
| Colorectum | B12 | rs2270655 | MMAA | G | C | 0.066 | 0.018 | 2.20E-13 | -0.020 | 0.064 | 0.750 |
| Colorectum | B12 | rs1141321 | MUT | C | T | 0.061 | 0.007 | 3.60E-26 | 0.014 | 0.031 | 0.655 |
| Colorectum | B12 | rs1801222 | CUBN | G | A | 0.110 | 0.007 | 0.00E+00 | 0.011 | 0.032 | 0.740 |
| Colorectum | B12 | rs117456053 | TCN1 | G | A | 0.160 | 0.026 | 1.90E-09 | 0.071 | 0.094 | 0.453 |
| Colorectum | B12 | rs34324219 | TCN1 | C | A | 0.210 | 0.007 | 0.00E+00 | 0.048 | 0.048 | 0.323 |
| Colorectum | B12 | rs34528912 | TCN1 | T | C | 0.170 | 0.021 | 2.10E-15 | -0.022 | 0.067 | 0.739 |
| Colorectum | B12 | rs56077122 | CUBN/TRDMT1 | A | C | 0.087 | 0.009 | 4.80E-21 | 0.005 | 0.035 | 0.890 |
| Colorectum | B12 | rs41281112 | CLYBL | C | T | 0.170 | 0.020 | 8.90E-35 | -0.052 | 0.086 | 0.540 |
| Colorectum | B12 | rs3742801 | ABCD4 | T | C | 0.045 | 0.009 | 1.70E-13 | 0.030 | 0.032 | 0.341 |
| Colorectum | B12 | rs2336573 | CD320 | T | C | 0.320 | 0.007 | 0.00E+00 | 0.116 | 0.094 | 0.214 |
| Colorectum | B12 | rs602662 | FUT2 | A | G | 0.160 | 0.007 | 0.00E+00 | 0.021 | 0.030 | 0.479 |
| Colorectum | B12 | rs1131603 | TCN2 | C | T | 0.190 | 0.017 | 0.00E+00 | 0.060 | 0.053 | 0.260 |
| Colorectum | B6 | rs4654748 | ALPL | T | C | 1.450 | 0.280 | 8.30E-18 | -0.028 | 0.030 | 0.347 |
| Colorectum | Folate | rs1801133 | MTHFR | G | A | 0.096 | 0.008 | 0.00E+00 | -0.019 | 0.035 | 0.583 |
| Colorectum | Folate | rs652197 | FOLR3 | C | T | 0.069 | 0.011 | 1.40E-12 | 0.003 | 0.038 | 0.929 |
| Esophagus | B12 | rs2270655 | MMAA | G | C | 0.066 | 0.018 | 2.20E-13 | 0.194 | 0.220 | 0.379 |
| Esophagus | B12 | rs1141321 | MUT | C | T | 0.061 | 0.007 | 3.60E-26 | -0.129 | 0.105 | 0.220 |
| Esophagus | B12 | rs1801222 | CUBN | G | A | 0.110 | 0.007 | 0.00E+00 | 0.004 | 0.110 | 0.970 |
| Esophagus | B12 | rs117456053 | TCN1 | G | A | 0.160 | 0.026 | 1.90E-09 | -0.440 | 0.317 | 0.166 |
| Esophagus | B12 | rs34324219 | TCN1 | C | A | 0.210 | 0.007 | 0.00E+00 | 0.232 | 0.166 | 0.163 |
| Esophagus | B12 | rs34528912 | TCN1 | T | C | 0.170 | 0.021 | 2.10E-15 | 0.110 | 0.227 | 0.629 |
| Esophagus | B12 | rs56077122 | CUBN/TRDMT1 | A | C | 0.087 | 0.009 | 4.80E-21 | 0.084 | 0.118 | 0.478 |
| Esophagus | B12 | rs41281112 | CLYBL | C | T | 0.170 | 0.020 | 8.90E-35 | 0.040 | 0.289 | 0.889 |
| Esophagus | B12 | rs3742801 | ABCD4 | T | C | 0.045 | 0.009 | 1.70E-13 | -0.238 | 0.110 | 0.030 |
| Esophagus | B12 | rs2336573 | CD320 | T | C | 0.320 | 0.007 | 0.00E+00 | 0.209 | 0.321 | 0.516 |
| Esophagus | B12 | rs602662 | FUT2 | A | G | 0.160 | 0.007 | 0.00E+00 | -0.078 | 0.103 | 0.449 |
| Esophagus | B12 | rs1131603 | TCN2 | C | T | 0.190 | 0.017 | 0.00E+00 | 0.166 | 0.180 | 0.356 |
| Esophagus | B6 | rs4654748 | ALPL | T | C | 1.450 | 0.280 | 8.30E-18 | -0.006 | 0.102 | 0.956 |
| Esophagus | Folate | rs1801133 | MTHFR | G | A | 0.096 | 0.008 | 0.00E+00 | 0.023 | 0.121 | 0.848 |
| Esophagus | Folate | rs652197 | FOLR3 | C | T | 0.069 | 0.011 | 1.40E-12 | -0.100 | 0.130 | 0.441 |
| Pancreas | B12 | rs2270655 | MMAA | G | C | 0.066 | 0.018 | 2.20E-13 | 0.016 | 0.133 | 0.904 |
| Pancreas | B12 | rs1141321 | MUT | C | T | 0.061 | 0.007 | 3.60E-26 | -0.131 | 0.065 | 0.043 |
| Pancreas | B12 | rs1801222 | CUBN | G | A | 0.110 | 0.007 | 0.00E+00 | -0.102 | 0.068 | 0.129 |
| Pancreas | B12 | rs117456053 | TCN1 | G | A | 0.160 | 0.026 | 1.90E-09 | -0.029 | 0.188 | 0.877 |
| Pancreas | B12 | rs34324219 | TCN1 | C | A | 0.210 | 0.007 | 0.00E+00 | -0.238 | 0.101 | 0.019 |
| Pancreas | B12 | rs34528912 | TCN1 | T | C | 0.170 | 0.021 | 2.10E-15 | 0.077 | 0.140 | 0.585 |
| Pancreas | B12 | rs56077122 | CUBN/TRDMT1 | A | C | 0.087 | 0.009 | 4.80E-21 | 0.048 | 0.073 | 0.514 |
| Pancreas | B12 | rs41281112 | CLYBL | C | T | 0.170 | 0.020 | 8.90E-35 | 0.241 | 0.175 | 0.169 |
| Pancreas | B12 | rs3742801 | ABCD4 | T | C | 0.045 | 0.009 | 1.70E-13 | 0.028 | 0.067 | 0.682 |
| Pancreas | B12 | rs2336573 | CD320 | T | C | 0.320 | 0.007 | 0.00E+00 | -0.287 | 0.196 | 0.144 |
| Pancreas | B12 | rs602662 | FUT2 | A | G | 0.160 | 0.007 | 0.00E+00 | -0.051 | 0.064 | 0.419 |
| Pancreas | B12 | rs1131603 | TCN2 | C | T | 0.190 | 0.017 | 0.00E+00 | -0.012 | 0.111 | 0.916 |
| Pancreas | B6 | rs4654748 | ALPL | T | C | 1.450 | 0.280 | 8.30E-18 | -0.014 | 0.063 | 0.826 |
| Pancreas | Folate | rs1801133 | MTHFR | G | A | 0.096 | 0.008 | 0.00E+00 | -0.048 | 0.074 | 0.522 |
| Pancreas | Folate | rs652197 | FOLR3 | C | T | 0.069 | 0.011 | 1.40E-12 | -0.116 | 0.079 | 0.142 |
| Stomach | B12 | rs2270655 | MMAA | G | C | 0.066 | 0.018 | 2.20E-13 | 0.114 | 0.134 | 0.396 |
| Stomach | B12 | rs1141321 | MUT | C | T | 0.061 | 0.007 | 3.60E-26 | 0.019 | 0.066 | 0.771 |
| Stomach | B12 | rs1801222 | CUBN | G | A | 0.110 | 0.007 | 0.00E+00 | -0.086 | 0.068 | 0.210 |
| Stomach | B12 | rs117456053 | TCN1 | G | A | 0.160 | 0.026 | 1.90E-09 | -0.169 | 0.200 | 0.398 |
| Stomach | B12 | rs34324219 | TCN1 | C | A | 0.210 | 0.007 | 0.00E+00 | 0.099 | 0.102 | 0.330 |
| Stomach | B12 | rs34528912 | TCN1 | T | C | 0.170 | 0.021 | 2.10E-15 | -0.114 | 0.143 | 0.423 |
| Stomach | B12 | rs56077122 | CUBN/TRDMT1 | A | C | 0.087 | 0.009 | 4.80E-21 | 0.079 | 0.074 | 0.285 |
| Stomach | B12 | rs41281112 | CLYBL | C | T | 0.170 | 0.020 | 8.90E-35 | -0.171 | 0.181 | 0.345 |
| Stomach | B12 | rs3742801 | ABCD4 | T | C | 0.045 | 0.009 | 1.70E-13 | 0.019 | 0.068 | 0.784 |
| Stomach | B12 | rs2336573 | CD320 | T | C | 0.320 | 0.007 | 0.00E+00 | -0.100 | 0.197 | 0.611 |
| Stomach | B12 | rs602662 | FUT2 | A | G | 0.160 | 0.007 | 0.00E+00 | -0.040 | 0.064 | 0.530 |
| Stomach | B12 | rs1131603 | TCN2 | C | T | 0.190 | 0.017 | 0.00E+00 | 0.206 | 0.112 | 0.065 |
| Stomach | B6 | rs4654748 | ALPL | T | C | 1.450 | 0.280 | 8.30E-18 | 0.033 | 0.063 | 0.605 |
| Stomach | Folate | rs1801133 | MTHFR | G | A | 0.096 | 0.008 | 0.00E+00 | -0.096 | 0.076 | 0.206 |
| Stomach | Folate | rs652197 | FOLR3 | C | T | 0.069 | 0.011 | 1.40E-12 | -0.003 | 0.081 | 0.968 |

EA indicates effect allele; NEA, non-effect allele; SE, standard error; SNP, single nucleotide polymorphism; VB, vitamin B.

**Supplementary Table 4**. Diseases and traits associated with genetic variants identified for folate, vitamin B6, and vitamin B12 at the genome-wide significance level

| **SNP** | **Chromosome** | **Effect allele** | **Diseases and traits** |
| --- | --- | --- | --- |
| Vitamin B6 |  |  |  |
| rs4654748 | 1 | T | Alkaline phosphatase (-) |
| Folate |  |  |  |
| rs1801133 | 1 | G | Homocysteine levels (-)  Diastolic blood pressure (-)  Mean corpuscular hemoglobin (-) |
| rs652197 | 11 | C | NA |
| Vitamin B12 |  |  |  |
| rs12272669 | 1 | A | NA |
| rs2270655 | 4 | G | NA |
| rs1141321 | 6 | C | NA |
| rs1801222 | 10 | G | Homocysteine levels (-) |
| rs34324219 | 11 | C | Self-reported pernicious anaemia (-)  Self-reported high cholesterol (+)  Alkaline phosphatase (-)  Cholelithiasis (+)  Crohns disease (+)  Sodium in urine (-)  Mean platelet volume (-)  Alcohol intake frequency (-)  Total cholesterol (+)  Treatment with simvastatin (+)  Bring up phlegm or sputum/mucus on most days (+)  Gamma glutamyl transferase (?)  Pediatric autoimmune diseases (?) |
| rs34528912 | 11 | T | NA |
| rs117456053 | 11 | G | NA |
| rs56077122 | 11 | A | NA |
| rs41281112 | 13 | C | NA |
| rs3742801 | 14 | T | NA |
| rs2336573 | 19 | T | NA |
| rs7788053 | 19 | A | NA |
| rs602662 | 19 | A | Lewis system Lea antigen (?) |
| rs1131603 | 22 | C | Cardiovascular disease prevalence (?) |

NA indicates not available or no associated disease or traits reported; SNP, single nucleotide polymorphism.

**Supplementary Figure 1.** Associations of genetically predicted higher serum folate and vitamin B12 with pernicious anemia (positive control outcome)

**Associations in different MR analysis models**

| **Exposure** | **Method** | **OR** | **95% CI** | **p** |
| --- | --- | --- | --- | --- |
| Folate | IVW-fixed effects | 0.36 | 0.15, 0.84 | 0.018 |
| B12 | IVW-random effects | 0.47 | 0.28, 0.81 | 0.006 |
| B12 | Weighted median | 0.44 | 0.28, 0.70 | 0.001 |
| B12 | MR-Egger | 0.92 | 0.44, 1.96 | 0.838 |

**Associations in leave-one-out analysis for vitamin B12**


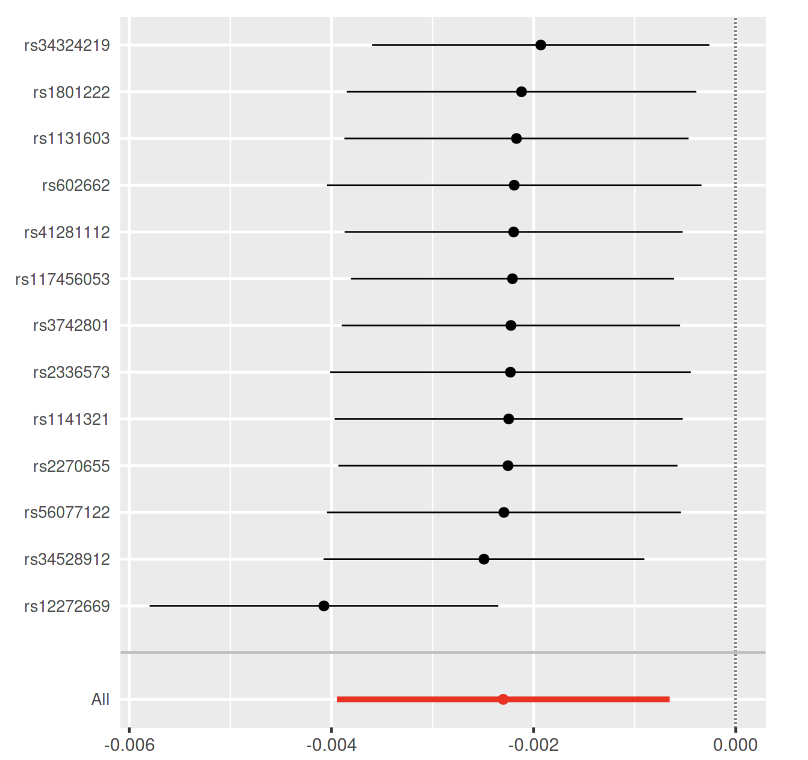


CI indicates confidence interval; IVW, inverse-variance weighted; OR, odds ratio; SD, standard deviation.

**Supplementary Figure 2.** Associations of genetically predicted higher serum folate and vitamin B12 with mean corpuscular volume (positive control outcome)

**Associations in different MR analysis models**

| **Exposure** | **Method** | **Change** | **95% CI** | **p** |
| --- | --- | --- | --- | --- |
| Folate | IVW-fixed effects | -0.12 | -0.16, -0.09 | <0.001 |
| B12 | IVW-random effects | -0.01 | -0.03, 0.01 | 0.200 |
| B12 | Weighted median | 0.00 | -0.01, 0.01 | 0.820 |
| B12 | MR-Egger | -0.01 | -0.05, 0.02 | 0.407 |

**Associations in leave-one-out analysis for vitamin B12**

**
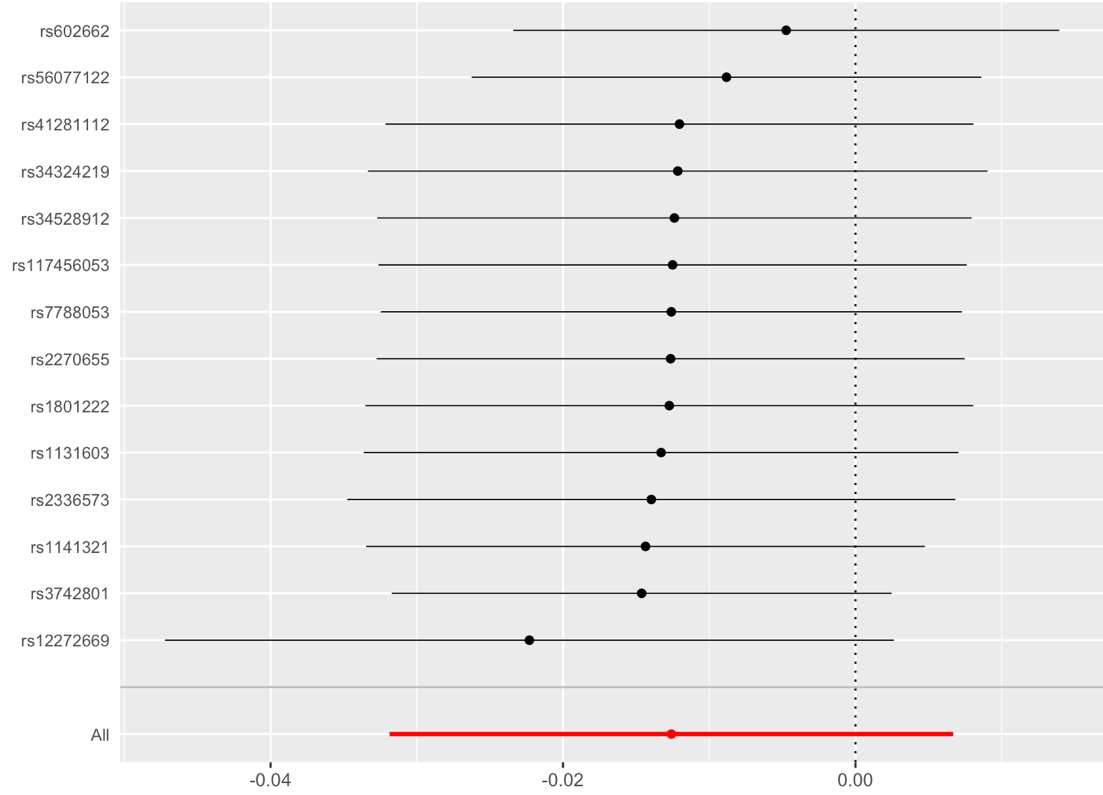
**

CI indicates confidence interval; IVW, inverse-variance weighted; OR, odds ratio; SD, standard deviation.

**Supplementary Figure 3.** Associations of genetically predicted higher serum vitamin B12 with any digestive system cancer and colorectal cancer in leave-one-out analysis based on UK Biobank

**Any digestive system cancer**

**
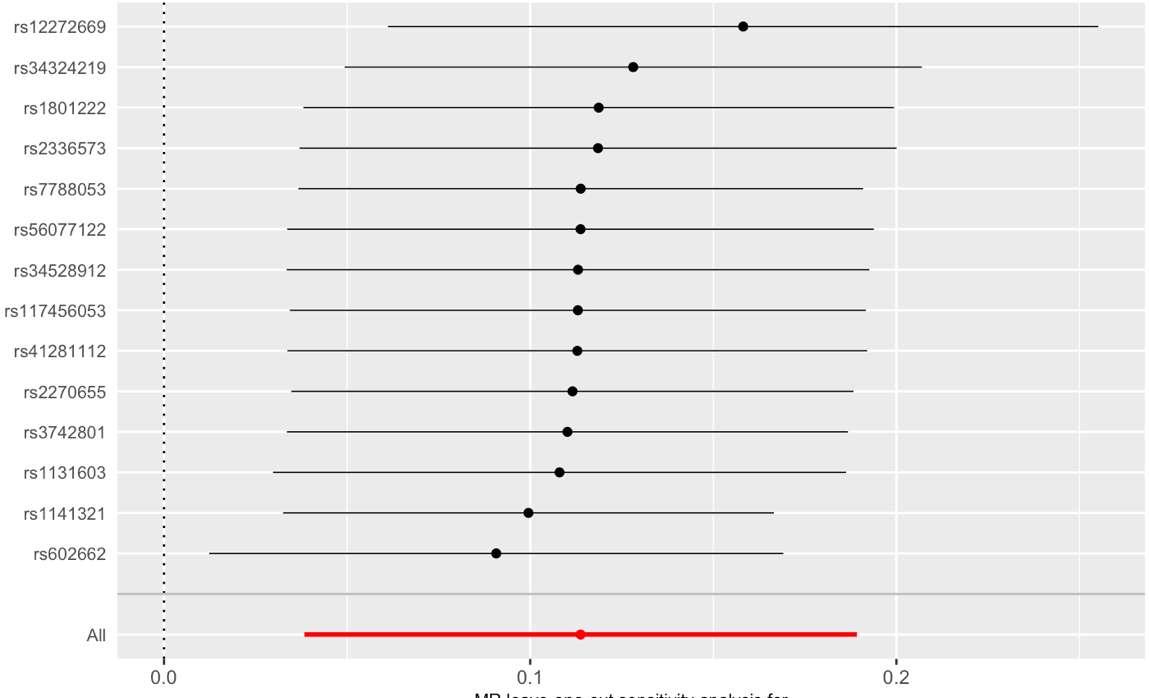
**

**Colorectal cancer**


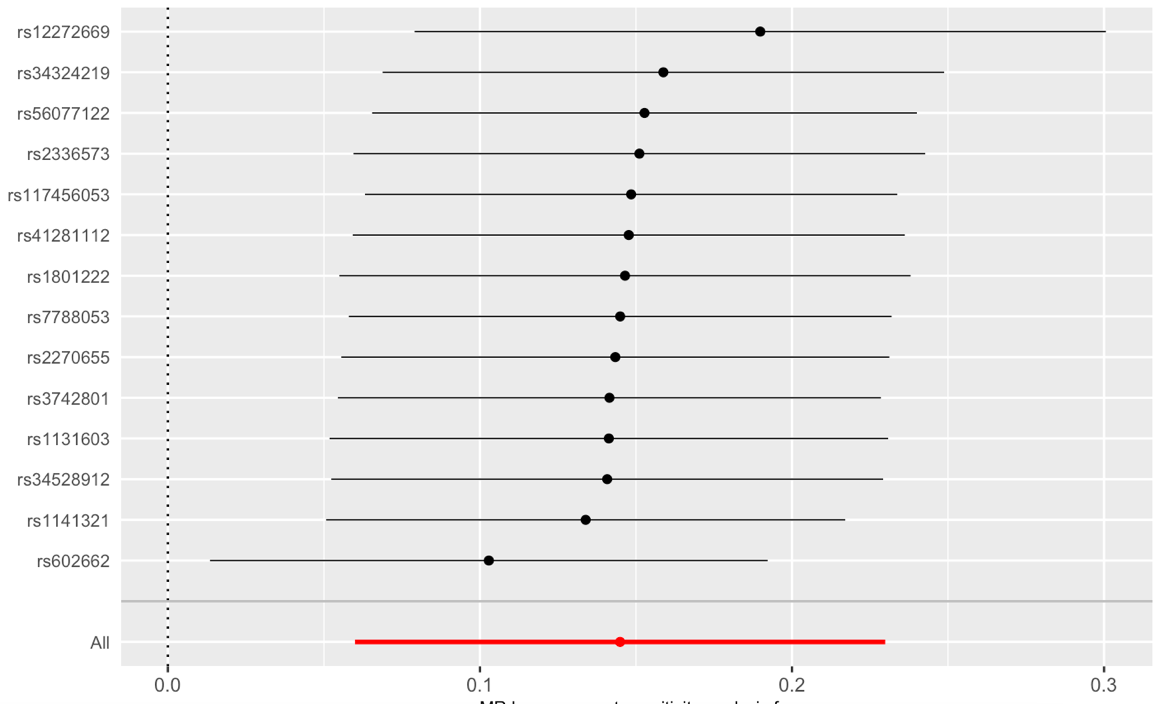

Supplement: Supplementary file 1 — Supporting materials [file 41416_2021_1383_MOESM1_ESM.docx]
